# Experimental data for a flow control valve

**DOI:** 10.1016/j.dib.2019.103892

**Published:** 2019-04-03

**Authors:** P. Rezazadeh, M. Bijankhan, A. Mahdavi Mazdeh

**Affiliations:** a Water Science and Engineering Dept., Imam Khomeini International University, Qazvin, Iran; b Ruhr-Universität Bochum, Fakultät für Geowissenschaften, Institut Geologie, Mineralogie und Geophysik, Germany

## Abstract

Experimental data presented in this paper include the flow measurements through cylindrical orifices and the discharge behavior of Mechanical Choked Orifice Plate, MCOP. The data information consists of detailed physical shapes of the devices as well as their application in pressurized networks. From the hydraulic point of view, discharge measurements with their associated differential pressures are presented. The data analyses are presented in “An Experimental study on a flow control device applicable in pressurized networks” Rezazadeh et al., 2019.

**Table:** Specifications table

Subject area	*Agriculture, Civil engineering*
More specific subject area	*Flow control, Flow measurement*
Type of data	*Tables*
How data was acquired	*Physical model of the cylindrical orifices and valves were constructed. Discharge values were recorded by a pre-calibrated orifice. The differential pressure was recorded by a Rosemount digital pressure transmitter; model 3051 Coplanar Pressure Transmitter whose total response time is* 100 ms*. Allowable differential pressure limit was in the range of ±2.*5 bar *with a total performance of ±0.15% of span.*
Data format	*Raw*
Experimental factors	*Discharge, pressure*
Experimental features	*Experiments were done in room temperature and using fresh water with low total dissolved solids*
Data source location	*Qazvin, Iran*
Data accessibility	*Data is with this article.*
Related research article	P. Rezazadeh, M. Bijankhan, A. Mahdavi Mazdeh, An Experimental study on a flow control device applicable in pressurized networks, Flow Meas. Instrum. (2019), https://doi.org/10.1016/j.flowmeasinst. 2019.01.017., [1].

**Table:** 

**Value of the data** • The data can be used to enhance the performance of MCOP; • Numerical models can be validated using the data published in this paper; • The presented data are useful to know about the Hydraulic behavior of the cylindrical orifices.

## 1 Data

Mechanical Choked Orifice Plate is a device to deliver an almost constant discharge in pressurized network. In this data article the experimental data of the flow through a cylindrical orifice is presented first. Then, the performance curves (Discharge and differential pressure data), of the tested MCOP devices are presented.

**Fig. 1:**
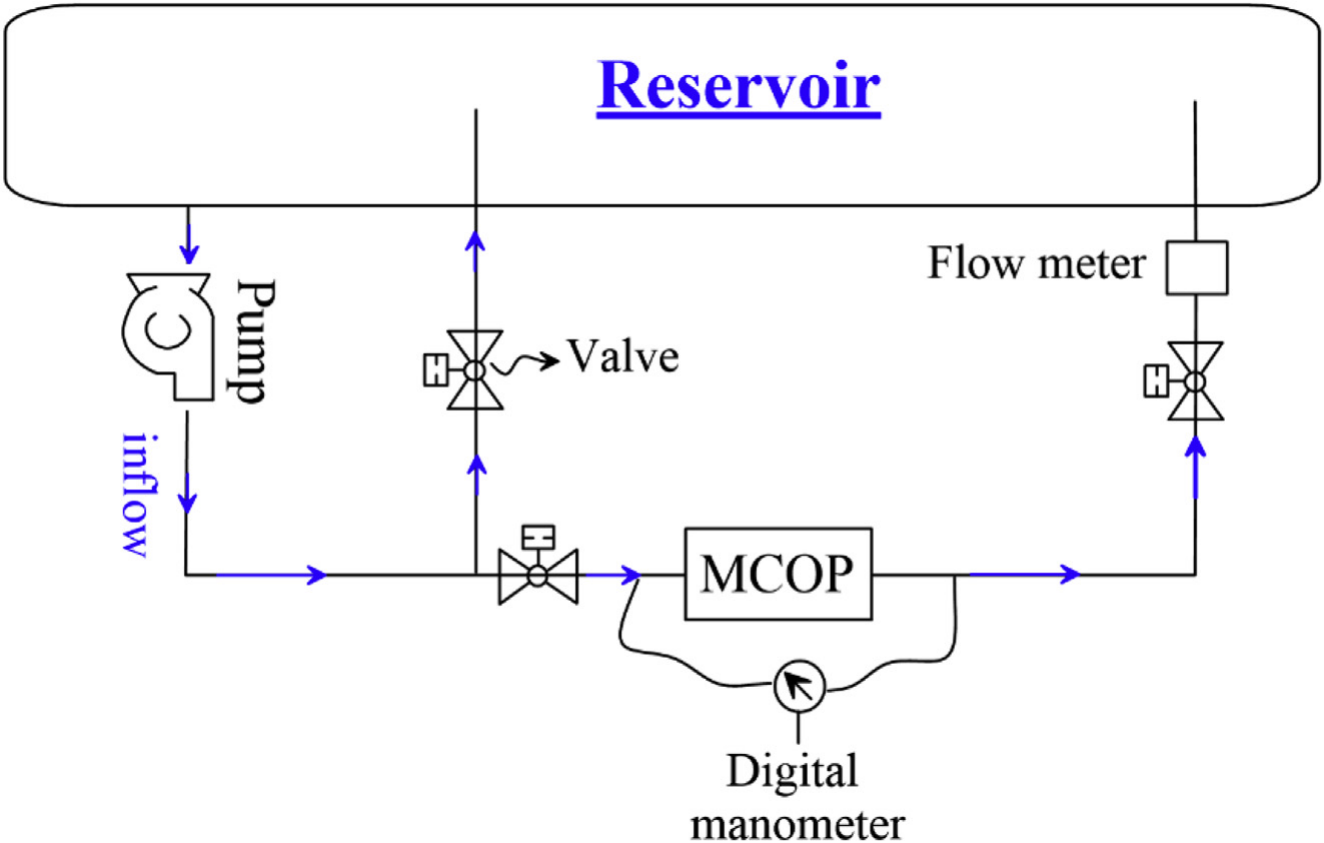
Schematic view of the experimental setup located at the hydraulic laboratory at IKIU, Qazvin, Iran.

**Fig. 2:**
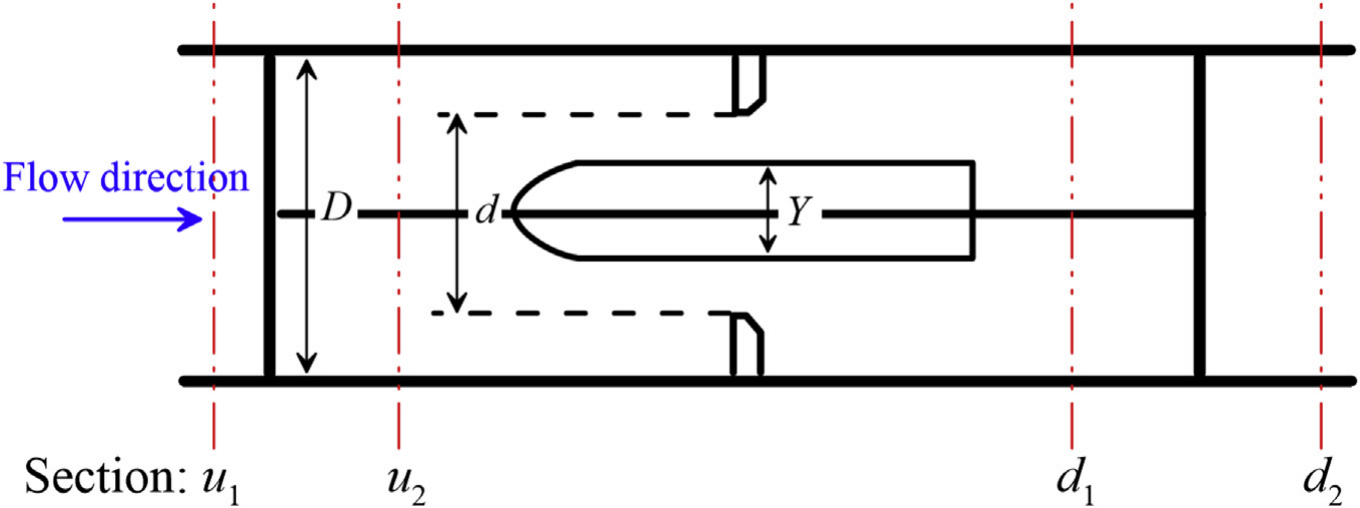
Schematic view of the cylindrical orifice device.

Fig. 1 is schematic view of the experimental setup to test the MCOP performance. Fig. 2 indicates the view of the cylindrical orifice device whose experimental data can be used to enhance the performance of MCOP.

The discharge values and the associated pressure values of the flow through cylindrical orifice are listed in Table 1.

**Table 1:** Detailed experimental data of the flow through cylindrical orifice.

*Y* (*mm*)	*P*u_1_ (*m*)	*pu*_2_ (*m*)	*pd*_1_ (*m*)	*pd*_2_ (*m*)	*Q* (*l*/*s*)
15.8	0.357	0.346	0.108	0.12	0.750
15.8	0.967	0.934	0.318	0.318	0.478
15.8	1.778	1.727	0.603	0.603	0.817
15.8	2.786	2.71	0.951	0.951	0.883
15.8	4	3.88	1.37	1.37	0.983
15.8	5.42	5.27	1.85	1.85	1.079
15.8	7.022	6.81	2.36	2.36	1.171
15.8	8.82	8.57	2.96	2.96	1.254
15.8	10.79	10.48	3.62	3.62	1.282
15.8	12.96	12.58	4.37	4.37	1.440
15.8	15.24	14.85	5.12	5.12	0.875
15.8	17.74	17.24	5.9	5.9	0.177
15.8	20.47	19.87	6.9	6.9	0.297
15.8	22.3	21.67	7.33	7.33	0.407
17.2	0.398	0.395	0.038	0.058	0.513
17.2	1.101	1.092	0.041	0.121	0.617
17.2	2.042	2.018	0.062	0.217	0.720
17.2	3.21	3.18	0.11	0.35	0.824
17.2	8.09	8.03	0.28	0.89	0.990
17.2	10.16	10.08	0.33	1.11	1.055
17.2	11.68	11.58	0.38	1.29	1.121
17.2	13.26	13.14	0.39	1.45	1.185
17.2	14.94	14.8	0.4	1.66	1.250
17.2	16.7	16.54	0.48	1.87	1.314
14.3	0.388	0.364	0.203	0.216	1.382
14.3	0.952	0.916	0.55	0.452	1.466
14.3	1.7	1.63	0.91	0.814	0.750
14.3	2.63	2.54	1.42	1.27	0.478
14.3	3.75	3.62	2.03	1.82	0.817
14.3	5.053	4.871	2.715	2.412	0.883
14.3	6.57	6.296	3.542	3.17	0.983
14.3	8.181	7.9	4.398	3.901	1.079
14.3	9.412	9.05	5.082	4.502	1.171
14.3	10.67	10.27	5.77	5.145	1.254
14.3	12.67	12.2	6.79	6.03	1.282
14.3	14.88	14.35	7.94	7.168	1.440
14.3	16.375	15.84	8.764	7.855	0.875
14.3	18.894	18.233	10.031	8.994	0.177
14.3	20.48	19.75	10.95	9.76	0.297
13	0.356	0.341	0.208	0.183	0.407
13	0.901	0.866	0.399	0.495	0.513
13	1.61	1.54	1.014	0.901	0.617
13	2.52	2.41	1.606	1.42	0.720
13	3.5	3.45	2.186	1.93	0.824
13	4.84	4.62	3.076	2.71	0.990
13	6.241	6.028	3.953	3.496	1.055
13	7.836	7.54	4.996	4.397	1.121
13	8.997	8.68	5.731	5.036	1.185
13	10.19	9.79	6.45	5.703	1.250
13	12.12	11.7	7.67	6.76	1.314
13	14.243	13.717	9.022	7.93	1.382
13	15.7	15.11	9.959	8.742	1.466
13	18.16	17.417	11.51	10.17	0.750
13	19.66	18.933	12.49	10.94	0.478
10.6	0.657	0.612	0.497	0.442	0.817
10.6	1.056	0.996	0.798	0.71	0.883
10.6	1.518	1.44	1.147	0.968	0.983
10.6	2.06	1.95	1.548	1.376	1.079
10.6	2.7	2.56	2.039	1.812	1.171
10.6	3.38	3.17	2.546	2.55	1.254
10.6	4.179	3.96	3.159	2.818	1.282
10.6	4.99	4.727	3.751	3.36	1.440
10.6	2.07	1.97	1.56	1.39	0.875
10.6	2.9	2.6	1.437	0.973	0.177
10.6	6.92	6.56	5.2	4.85	0.297
10.6	8.52	8.069	6.4	5.74	0.407
10.6	10.21	9.72	7.69	6.87	0.513
10.6	12.12	11.48	9.1	8.13	0.617
10.6	14.08	13.34	10.53	9.44	0.720
10.6	16.24	15.47	12.16	10.87	0.824
10.6	18.39	17.4	13.7	12.34	0.990
10.6	6.86	6.5	5.16	4.61	1.055
10.6	0.289	0.272	0.2022	0.185	1.121
10.6	0.795	0.753	0.58	0.54	1.185
10.6	1.462	1.399	1.073	0.999	1.250
10.6	2.31	2.2	1.707	1.589	1.314
10.6	3.333	3.17	2.476	2.303	1.382
10.6	4.5	4.269	3.342	3.098	1.466
10.6	5.814	5.512	4.319	4.013	0.750
10.6	8.38	7.944	6.22	5.76	0.478
10.6	9.5	8.99	7.05	6.52	0.817
10.6	10.82	10.18	8.057	7.481	0.883
10.6	12	11.39	8.92	8.25	0.983
10.6	13.32	12.69	9.85	9.14	1.079
10.6	14.63	13.93	10.78	10.03	1.171
10.6	16.14	15.35	11.84	11.028	1.254
10.6	18.17	17.32	13.32	12.4	1.282

Detailed experimental data of MCOP obtained based on the initial design method proposed by Rezazadeh et al. [1] for *Q* = 0.4 l/s (with 1≤Δ*p* (*m*)≤7), *Q* = 0.6 l/s (with 1≤Δ*p* (*m*)≤7), and *Q* = 0.6 l/s (with 2.6≤Δ*p* (*m*)≤20) are listed in Table 2, Table 3, and Table 4 respectively.

**Table 2:** Detailed experimental data of MCOP obtained based on the initial design method proposed by Rezazadeh et al. [1] for the case of *Q* = 0.4 l/s (with 1≤Δ*p* (*m*)≤7).

*P*u_1_ (*m*)	*pu*_2_ (*m*)	*pd*_1_ (*m*)	*pd*_2_ (*m*)	*Q* (*l*/*s*)
0.39	0.374	0.28	0.262	0.189
0.716	0.699	0.476	0.456	0.261
1.146	1.126	0.682	0.673	0.323
1.83	1.8	0.68	0.71	0.333
2.59	2.57	0.77	0.77	0.347
3.43	3.39	0.95	0.91	0.376
4.35	4.32	1.05	1.04	0.407
5.43	5.4	1.09	1.14	0.425
6.62	6.56	1.19	1.21	0.441
7.82	7.78	1.26	1.38	0.477
9.1	9.03	1.57	1.64	0.520
10.36	10.27	2	2.1	0.584

**Table 3:** Detailed experimental data of MCOP obtained based on the initial design method proposed by Rezazadeh et al. [1] for the case of *Q* = 0.6 l/s (with 1≤Δ*p* (*m*)≤7).

*P*u_1_ (*m*)	*pu*_2_ (*m*)	*pd*_1_ (*m*)	*pd*_2_ (*m*)	*Q* (*l*/*s*)
0.677	0.661	0.455	0.443	0.264
1.091	1.062	0.702	0.69	0.332
1.601	1.56	0.972	0.963	0.393
2.228	2.177	1.207	1.211	0.445
3.02	2.98	1.29	1.348	0.475
3.98	3.93	1.41	1.47	0.491
4.99	4.93	1.615	1.64	0.523
6.13	6.07	1.79	1.82	0.545
7.33	7.27	1.859	1.97	0.572
8.66	8.58	2.03	2.16	0.601
9.83	9.73	2.55	2.695	0.667
11	10.85	3.2	3.32	0.744
5.66	5.67	1.76	1.8	0.530
6.75	6.67	1.852	1.9	0.560
8.02	7.94	1.93	2.07	0.583

**Table 4:** Detailed experimental data of MCOP obtained based on the initial design method proposed by Rezazadeh et al. [1] for the case of *Q* = 0.6 l/s (with 2.6≤Δ*p* (*m*)≤20).

*P*u_1_ (*m*)	*pu*_2_ (*m*)	*pd*_1_ (*m*)	*pd*_2_ (*m*)	*Q* (*l*/*s*)
0.939	0.878	0.433	0.385	0.271
1.454	1.377	0.635	0.569	0.325
2.088	1.985	0.858	0.768	0.371
2.82	2.69	1.05	0.95	0.412
3.67	3.523	1.26	1.15	0.450
4.58	4.46	1.44	1.34	0.490
5.61	5.43	1.71	1.6	0.532
6.75	6.58	1.85	1.78	0.562
8.02	7.86	2.07	1.96	0.591
9.4	9.28	2.27	2.15	0.617
10.88	10.75	2.48	2.37	0.642
12.41	11.99	2.62	2.48	0.669
14.05	13.94	2.93	2.75	0.705
15.79	15.65	3.17	3.03	0.727
17.68	17.55	3.27	3.1	0.748
19.69	19.58	3.23	3.26	0.755
22.01	21.89	3.15	3.08	0.747
23.85	23.69	3.66	3.71	0.814

Finally, experimental data of MCOP obtained based on the enhanced design method proposed by Rezazadeh et al. [1] for the cases of *Q* = 0.4 l/s (with 1≤Δ*p* (*m*)≤7), *Q* = 0.6 l/s (with 1≤Δ*p* (*m*)≤7), and *Q* = 0.6 l/s (with 2.6≤Δ*p* (*m*)≤20) are presented in Table 5, Table 6, and Table 7 respectively.

**Table 5:** Detailed experimental data of MCOP obtained based on the enhanced design method proposed by Rezazadeh et al. [1] for the case of *Q* = 0.4 l/s (with 1≤Δ*p* (*m*)≤7).

*Pu*_1_ (m)	*pu*_2_ (m)	*pd*_1_ (m)	*pd*_2_ (m)	*Q* (*l*/*s*)
0.788	0.773	0.517	0.494	0.282
1.259	1.228	0.773	0.748	0.347
1.86	1.8	0.999	0.964	0.394
2.77	2.73	0.82	0.91	0.381
3.77	3.73	0.8	0.82	0.364
4.78	4.75	0.81	0.93	0.389
5.97	5.92	0.81	0.99	0.395
7.25	7.21	0.87	0.96	0.393
8.55	8.5	0.91	1.08	0.418
9.73	9.67	1.35	1.48	0.501
10.88	10.82	1.99	2.06	0.592
12.7	12.62	3.1	3.06	0.726

**Table 6:** Detailed experimental data of MCOP obtained based on the enhanced design method proposed by Rezazadeh et al. [1] for the case of *Q* = 0.6 l/s (with 1≤Δ*p* (*m*)≤7).

*Pu*_1_ (m)	*pu*_2_ (m)	*pd*_1_ (m)	*pd*_2_ (m)	*Q* (*l*/*s*)
0.75	0.7	0.592	0.555	0.294
1.177	1.129	0.915	0.865	0.367
1.67	1.61	1.28	1.216	0.439
2.23	2.16	1.677	1.595	0.510
2.84	2.75	2.116	1.966	0.581
3.59	3.49	2.602	2.41	0.644
4.12	4.03	2.56	2.54	0.659
4.75	4.66	2.53	2.47	0.656
6.35	5.83	2.96	2.36	0.637
5.58	5.5	2.26	2.31	0.624
6.63	6.53	2.16	2.21	0.612
7	6.94	2.13	2.18	0.607
7.76	7.71	2	2.09	0.598
8.5	8.44	2.04	2.11	0.596
9.15	9.08	2.25	2.26	0.620
10.25	10.16	2.82	2.94	0.707
11.43	11.32	3.48	3.61	0.784
12.67	12.56	4.17	4.33	0.859

**Table 7:** Detailed experimental data of MCOP obtained based on the enhanced design method proposed by Rezazadeh et al. [1] for the case of *Q* = 0.6 l/s (with 2.6≤Δ*p* (*m*)≤20).

*pu*_1_ (m)	*pu*_2_ (m)	*pd*_1_ (m)	*pd*_2_ (m)	*Q* (*l*/*s*)
0.476	0.444	0.324	0.29	0.199
1.282	1.188	0.802	0.702	0.343
2.12	1.98	1.29	1.15	0.437
3.11	2.92	1.91	1.68	0.531
3.96	3.71	2.3	2.04	0.583
5.28	5.14	2.5	2	0.581
5.64	5.71	1.64	1.65	0.589
7.21	6.99	2.03	2.08	0.609
7.93	7.74	1.56	1.88	0.568
8.62	8.46	1.57	1.93	0.573
9.35	9.22	1.67	2	0.582
10.09	9.99	1.88	2.01	0.589
10.9	10.8	2.21	2.06	0.593
11.72	11.65	2.3	2.06	0.590
12.66	12.58	2.16	2.01	0.580
13.57	13.5	2.06	1.95	0.570
14.51	14.45	1.96	1.9	0.566
15.49	15.4	1.85	1.85	0.558
16.46	16.35	1.75	1.82	0.551
17.51	17.45	1.64	1.77	0.542
18.54	18.46	1.55	1.72	0.534
19.69	19.59	1.37	1.53	0.507
20.71	20.65	1.47	1.55	0.516
21.69	21.63	1.86	1.77	0.551
22.59	22.48	2.16	2.08	0.593

## 2 Experimental design, materials and methods

Experimental setup used in this data article to investigate the hydraulic behavior of the Mechanical Choked Orifice Plate, MCOP, is located at the hydraulic laboratory of the Water Sciences and Engineering Dept., Imam Khomeini International University, IKIU, Qazvin, Iran (Fig. 1).

The experimental setup was circulated by a reservoir and a pipe network. All tests were done in room temperature of 20–22. A variable speed centrifugal pump supplied the system by a maximum discharge of 2 l/s and shut off head of about 30 *m*. The differential pressure was recorded thanks to a Rosemount digital pressure transmitter; model 3051 Coplanar Pressure Transmitter whose total response time is 100 ms. Allowable differential pressure limit was in the range of ±2.5 bar with a total performance of ±0.15% of span.

An orifice was used to read the discharge. In this regard, the differential pressure through the orifice was recorded. Then, the associated mass flow was measured by weighing the outflow in a given time period. Finally, the calibrated discharge-differential pressure curve was used to read the flow rate with accuracy of ±1%.

Experiments were performed to develop a discharge formula for the cylindrical orifice flow. As shown in Fig. 2, a cylindrical orifice consists of a column shaped obstacle of diameter *Y* installed in an orifice with the diameter of *d*. To test the hydraulic behavior of the cylindrical orifice, different cylinder diameters, *Y*, were installed separately in an orifice of a given diameter of *d* = 18.5°mm with a main pipe of the diameter of *D* = 25.4°mm. Flow rate was fixed by adjusting the pump speed and the associated differential pressures, Δ*p*, were recorded using the digital manometer. As indicated in Fig. 2, the pressures were recorded in 4 sections, i.e. *u*_1_: an upstream section, *u*_2_: a section just after the entrance bracket, *d*_1_: a section just after the cylinder (or float when a MCOP was installed), *d*_2_: a section after the device.

The second series of the experiments were performed on the initial and enhanced designs of MCOP devices. Note that the float dimensions for initial and enhanced designs can be found in Rezazadeh et al. [1]. The control valves of different hydraulic characteristics, i.e. *Q* = 0.4 l/s (with 1≤Δ*p* (*m*)≤7), *Q* = 0.6 l/s (with 1≤Δ*p* (*m*)≤7), and *Q* = 0.6 l/s (with 2.6≤Δ*p* (*m*)≤20), were considered. In each experiment the pressure through the pipe network was incrementally increased by increasing the pump speed. Then the flow rate and pressure through the MCOP devices were recorded.
