# Supplementary material for: Experimental data for a flow control valve
Source: Data Brief. 2019 Apr 3;24:103892. doi: 10.1016/j.dib.2019.103892 (PMC6462793; doi:10.1016/j.dib.2019.103892)
Supplement: Multimedia component 1 [file mmc1.pdf]

Conflict of Interest and Authorship Conformation Form

Please check the following as appropriate:

- o All authors have participated in (a) conception and design, or analysis and interpretation of the data; (b) drafting the article or revising it critically for important intellectual content; and (c) approval of the final version.

- o This manuscript has not been submitted to, nor is under review at, another journal or other publishing venue.

Author's name Affiliation

Parisa Rezazadeh, Graduated Msc. Student, Water science and engineering Dept., Imam Khomeini International University, Qazvin, Iran.

Mohammad Bijankhan, Assistant Professor, Water science and engineering Dept., Imam Khomeini International University, Qazvin, Iran.

Ali Mahdavi Mazdeh, Assistant Professor, Water science and engineering Dept., Imam Khomeini International University, Qazvin, Iran., Ruhr-Universitaet Bochum, Fakultät für Geowissenschaften, Institut Geologie, Mineralogie und Geophysik (Visiting Professor) (Corresponding author)

Ali Mahdavi Mazdeh 10 April 2019.
